# Coupling Causal Inference and Cross-Modal Recalibration: A Unified Framework for Adaptive Multisensory Perception

**DOI:** 10.3390/biology15050376

**Published:** 2026-02-25

**Authors:** Jing Liu, Chu-Chung Huang, Fu Zeng

**Affiliations:** 1 Key Laboratory of Brain Functional Genomics (Ministry of Education), East China Normal University, Shanghai 200062, China; 52193200027@stu.ecnu.edu.cn (J.L.); czhuang@psy.ecnu.edu.cn (C.-C.H.); 2 Institute of Brain and Education Innovation, School of Psychology and Cognitive Science, East China Normal University, Shanghai 200062, China; 3 NYU-ECNU Institute of Brain and Cognitive Science, New York University Shanghai, Shanghai 200122, China

**Keywords:** multisensory perception, Bayesian causal inference, cross-modal, recalibration, neural plasticity

## Abstract

Perception in natural environments relies on the integration of information from multiple sensory modalities. Although combining sensory cues can improve perceptual accuracy, sensory signals are inherently noisy and do not always arise from a common source. As a result, the brain must continuously determine whether multisensory signals should be integrated or segregated, while also adapting sensory representations when discrepancies persist over time. Multisensory causal inference provides a principled framework of how the brain infers the causal structure underlying sensory inputs and flexibly arbitrates between integration and segregation. In parallel, extensive behavioral work has shown that prolonged exposure to cross-modal conflicts induces multisensory recalibration, leading to persistent changes in unisensory and multisensory perception. Despite progress in both areas, the relationship between causal inference and recalibration has remained unclear. In this review, we synthesize behavioral, computational, and neurophysiological evidence to argue that causal inference and recalibration form a coupled adaptive system operating across distinct time scales. We propose that causal inference constrains whether sensory discrepancies drive recalibration, whereas recalibration reshapes sensory representations and expectations, thereby influencing future causal judgments.

## Simple Summary

We experience the world by combining information from sight, hearing, touch, and balance. This usually helps us perceive more accurately, but the senses are noisy and sometimes describe different events. The brain therefore faces two linked challenges. First, it must decide, moment by moment, whether signals from different senses come from the same source and should be combined, or from different sources and should be kept separate. Second, when a mismatch between senses happens repeatedly, the brain can gradually “re-tune” how it interprets one or more senses so that the conflict becomes smaller in the future. This review explains evidence for both processes across several everyday situations, such as judging where a sound comes from when sight and sound disagree, feeling that a fake hand is part of your body when sight and touch match, and estimating self-motion when visual and vestibular cues conflict. We argue that the decision about “same source or different sources” likely helps control when recalibration happens, while recalibration can also reshape future decisions. Understanding this link may improve approaches to training, rehabilitation, and virtual reality design.

## 1. Introduction

Natural sensory environments are dynamic and often ambiguous. Signals from different sensory modalities are rarely perfectly aligned in space or time and vary in reliability across contexts [1,2,3]. To construct coherent percepts under such conditions, the brain must not only extract information from individual sensory channels but also infer how signals across modalities relate to one another.

A large body of work on multisensory integration has shown that observers often combine sensory cues in a reliability-weighted manner, approaching statistically optimal performance when discrepancies are small [4,5,6,7,8,9,10]. These findings motivated probabilistic accounts of perception that frame sensory processing as inference under uncertainty. However, sensory signals in the natural world do not always originate from a single source. When discrepancies between modalities become large, mandatory fusion can be detrimental, leading to systematic perceptual errors [11,12].

Multisensory causal inference formalizes this problem by proposing that the perceptual system evaluates alternative hypotheses about whether sensory signals arise from a common source or from separate sources, and adjusts perceptual estimates accordingly [13,14]. Behavioral evidence from spatial, temporal, bodily, and self-motion domains shows that multisensory interactions vary systematically with cue discrepancy, reliability, and prior experience, supporting the idea that inferred causal structure plays a central role in multisensory perception [15,16,17,18,19,20,21,22,23,24].

However, perception is shaped not only by moment-to-moment inference but also by experience accumulated over longer time scales. When discrepancies between modalities persist, the perceptual system often undergoes multisensory recalibration, gradually adjusting the mapping between sensory signals and perceptual variables [11,12,25,26]. Classic examples include the ventriloquism aftereffect [27,28], proprioceptive drift in the rubber hand illusion [29,30], and visual–vestibular recalibration during self-motion perception [31,32]. These phenomena indicate that multisensory processing is inherently adaptive, continuously incorporating environmental statistics into internal representations.

Traditionally, multisensory recalibration has been studied as a form of perceptual learning or adaptation, often characterized in terms of changes in sensory likelihoods or prior expectations [33,34,35]. In many cases, recalibration has been treated as a direct consequence of repeated exposure to discrepant sensory inputs, without explicit consideration of how the brain interprets the causal origin of these discrepancies. This raises a critical question: if two sensory signals are inferred to arise from different sources, why should their relationship be recalibrated at all? Conversely, when signals are consistently inferred to share a common cause despite small discrepancies, recalibration may be a rational strategy for improving future perceptual accuracy [26,32].

These considerations suggest that multisensory recalibration does not operate independently of causal inference. Small conflicts that support a high posterior probability of a common cause may promote integration and, with repeated exposure, drive recalibration [22,24,36,37]. In contrast, large conflicts favoring segregation may lead discrepancies to be attributed to distinct sources, thereby limiting or preventing learning [38,39]. From this perspective, causal inference determines not only whether sensory cues are integrated at a given moment but also whether discrepancies are treated as errors that warrant updating internal sensory representations [40,41].

In this review, we first summarize the principles and empirical evidence underlying multisensory causal inference, emphasizing how inferred causal structure modulates moment-to-moment integration. We then review behavioral and neural mechanisms of multisensory recalibration. Finally, we discuss how causal inference and recalibration may be linked within a unified framework of adaptive multisensory perception.

## 2. Multisensory Causal Inference

### 2.1. Conceptual Framework

Multisensory causal inference addresses a fundamental problem in perception: determining whether signals from different sensory modalities originate from the same external event. This challenge arises because natural sensory environments are structured by multiple objects and events that can generate overlapping or conflicting sensory signals. Accurate perception therefore requires not only estimating sensory variables within individual modalities, but also inferring which signals should be bound together and which should be treated as independent.

This problem differs fundamentally from classic cue integration frameworks, which assume that all available sensory cues reflect a single underlying cause and focus on how they should be optimally weighted [5,6,7]. Although such models successfully account for multisensory integration when discrepancies are small, they offer no principled account of how perception should operate when sensory signals are inconsistent or potentially originate from distinct sources.

Multisensory causal inference provides a conceptual solution by explicitly considering alternative causal interpretations of the sensory scene [13,15]. Rather than assuming obligatory fusion, the perceptual system evaluates whether sensory signals are more likely to reflect a common cause or separate causes, and adjusts perceptual estimates accordingly. At the behavioral level, this framework predicts that multisensory interactions should vary systematically with the degree of sensory conflict [13,15,18,19,23,24,42]. Signals that are closely aligned in space and time tend to be integrated, whereas large discrepancies favor perceptual segregation. Importantly, transitions between integration and segregation are often gradual rather than categorical, giving rise to partial integration under intermediate conflicts. Such nonlinear response patterns motivate the need for computational models that go beyond mandatory fusion and explicitly account for uncertainty about causal structure [43].

### 2.2. Bayesian Causal Inference Models

The dominant computational account of multisensory causal inference is the Bayesian causal inference (BCI) framework [13,15]. A central feature of this framework is the explicit representation of uncertainty about the causal structure that gave rise to sensory signals, rather than assuming that cues should always be integrated.

In its simplest form, the model assumes that sensory observations from different modalities (e.g., visual and vestibular signals) can be generated either by a common cause (C=1) or by separate causes (C=2). Given sensory measurements, the observer combines sensory evidence with a prior belief about the likelihood of a common source to compute a posterior probability over causal structures. This inference can be expressed as:

(1) $$P\left(C=1\;|\;X_{vis},X_{ves}\right)=\frac{P\left(X_{vis},X_{ves}\;|\;C=1\right)P_{\mathrm{prior}}}{P\left(X_{vis},X_{ves}\;|\;C=1\right)P_{\mathrm{prior}}+p\left(X_{vis},X_{ves}\;|\;C=2\right)\left(1-P_{\mathrm{prior}}\right)}$$

where $X_{vis}$ and $X_{ves}$ denote sensory measurements, C indexes the causal structure, and $P_{\mathrm{prior}}$ reflects the prior probability of a common cause. Importantly, this posterior provides a graded estimate of common-cause belief, rather than enforcing a categorical decision.

The inferred causal structure then shapes how perceptual estimates are formed. Under the common-cause hypothesis, sensory cues are assumed to be linked through a shared latent variable, favoring cue integration. Under the separate-causes hypothesis, cues are treated as unrelated and are therefore segregated. Rather than committing to a single hypothesis, the observer may combine estimates associated with different causal structures. For example, a model-averaged estimate of vestibular self-motion can be written as:

(2) $${\hat{S}}_{ves}=P\left(C=1\;|\;X_{ves},X_{vis}\right){\hat{S}}_{ves,C=1}+P\left(C=2\;|\;X_{ves},X_{vis}\right){\hat{S}}_{ves,C=2}$$

where ${\hat{S}}_{ves,C=1}$ and ${\hat{S}}_{ves,C=2}$ denote the vestibular estimates under the common-cause and separate-causes hypotheses, respectively. Because posterior causal probabilities are nonlinear functions of sensory inputs, the resulting perceptual estimates are likewise nonlinear, giving rise to characteristic transitions between integration and segregation as cue discrepancy increases.

Beyond providing a descriptive account of multisensory behavior, the BCI framework specifies a normative solution to two coupled inferential problems: identifying the most likely causal structure underlying sensory signals and estimating latent perceptual variables conditional on that structure [13]. These computations jointly explain why multisensory integration is robust for small discrepancies, attenuated for intermediate conflicts, and largely suppressed when segregation is favored.

**Figure 1:**
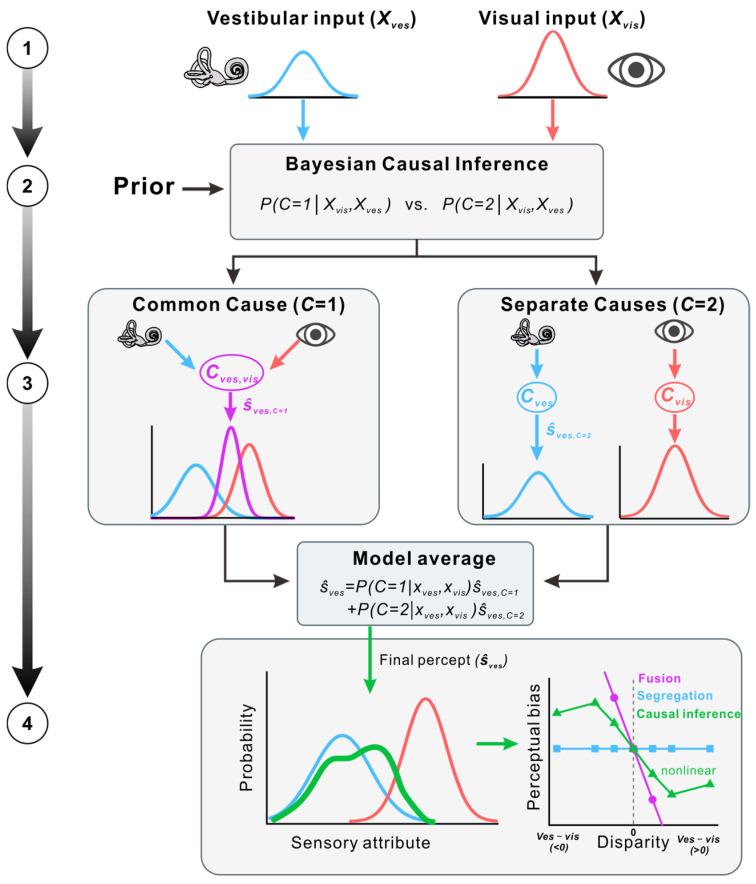
Computational framework of Bayesian causal inference in multisensory perception. (①) Individual sensory signals, such as vestibular $X_{vis}$ and visual $X_{ves}$ cues, are represented as probability distributions based on their reliability. (②) By combining sensory evidence with prior beliefs ($P_{\mathrm{prior}}$), the model computes the posterior probability that the cues arise from a common cause $P\left(C=1\;|\;X_{vis},X_{ves}\right)$ versus separate causes $P\left(C=2\;|\;X_{vis},X_{ves}\right)$. (③) If a common cause is inferred (C=1), cues are integrated to form a fused estimate; if separate causes are inferred (C=2), modality-specific estimates are maintained. (④) A final percept can be formed by model averaging, combining the C=1 and C=2 estimates weighted by their posterior probabilities. This computation produces a characteristic nonlinear dependence of perceptual bias on cue disparity, reflecting graded transitions between integration and segregation.

BCI models have been shown to account for a wide range of multisensory phenomena, including audiovisual spatial localization, temporal order judgments, speech perception, and visual–vestibular self-motion perception [13,15,18,19,20,23,24,44]. As such, Bayesian causal inference provides a unifying computational framework for understanding how integration and segregation emerge flexibly from uncertainty about the structure of the environment (Figure 1).

### 2.3. Behavioral Evidence for Multisensory Causal Inference

Behavioral studies provide strong evidence that humans and non-human primates perform causal inference during multisensory perception. One of the most extensively studied examples comes from audiovisual spatial localization, where interactions between vision and audition produce a continuous spectrum of perceptual outcomes [13,17,20,21,36,42]. Classic demonstrations such as the ventriloquist illusion show that when auditory and visual stimuli are spatially proximal, perceived sound location is strongly biased toward the visual signal. As spatial discrepancy increases, this cross-modal attraction weakens and can eventually give way to perceptual segregation [3,5].

Crucially, the transition from integration to segregation is not abrupt. Instead, behavioral responses change gradually, with partial integration often observed at intermediate disparities. Such nonlinear patterns are inconsistent with forced-fusion models, which predict obligatory integration regardless of cue discrepancy [43]. Bayesian causal inference models account for these observations by proposing that observers infer whether auditory and visual signals are causally linked. When signals are inferred to originate from a common source—typically at small spatial offsets—sensory cues are integrated. As evidence for separate causes increases, integration is progressively reduced or abolished [13,21,36,42,45].

Causal inference principles extend beyond spatial perception to temporal processing. In temporal numerosity judgment tasks, observers report the number of briefly presented flashes and beeps [46,47]. When auditory and visual event counts are mismatched, observers often experience the sound-induced flash illusion, in which additional beeps lead to the perception of extra flashes [15,48]. These effects are well explained by Bayesian causal inference models, which posit that observers infer whether auditory and visual events arise from a common temporal cause [15]. Integration occurs when temporal discrepancies fall within an inferred window of simultaneity, which depends not only on physical asynchrony but also on temporal acuity and prior expectations, underscoring the probabilistic nature of temporal binding [43].

Evidence for causal inference has also been reported in multisensory processes underlying body ownership [29,49,50,51,52]. In the rubber hand illusion, synchronous visual and tactile stimulation can induce a sense of ownership over an artificial limb, accompanied by proprioceptive drift toward the seen hand [24,29,53,54]. Bayesian causal inference models capture both the emergence of this illusion and its boundary conditions [24]. As spatial separation between the real and artificial hand increases, the inferred probability of a common cause decreases, leading to a breakdown of the illusion and a reduction in proprioceptive drift [53,55]. Importantly, similar body ownership illusions have been observed in non-human primates, and their behavioral responses are likewise consistent with causal inference accounts [56,57].

Further support for causal inference comes from self-motion perception, where the nervous system must determine whether changes in retinal input arise from self-generated movement or from motion in the external environment [18,19,22]. In heading perception tasks, observers judge self-motion direction based on visual optic flow and vestibular signals. Behavioral studies show that consistent visual and vestibular cues are integrated in a reliability-weighted manner, whereas large conflicts promote segregation of object motion from self-motion signals. This flexible arbitration between integration and segregation is well captured by Bayesian causal inference models and has been demonstrated in both humans [22] and non-human primates [18,19]. These findings highlight the role of causal inference in distinguishing self-related from externally generated sensory events.

Together, behavioral evidence from spatial, temporal, bodily, and self-motion domains support the view that multisensory interactions are governed by probabilistic inferences about causal structure, rather than by fixed or mandatory integration rules. Importantly, these behavioral signatures imply the existence of neural mechanisms capable of representing sensory uncertainty, evaluating causal structure, and dynamically shaping perceptual estimates over time. In the next section, we review neurophysiological and neuroimaging evidence that provides insight into how multisensory causal inference may be implemented in the brain.

### 2.4. Neural Mechanisms of Multisensory Causal Inference

Early work on the neural basis of multisensory causal inference relied largely on non-invasive neuroimaging in humans, converging on a distributed and hierarchical cortical architecture. Using audiovisual spatial localization, Rohe and Noppeney related regional functional magnetic resonance imaging (fMRI) activity to variables derived from Bayesian causal inference models and revealed a clear functional gradient along the cortical hierarchy [16]. Early sensory cortices primarily reflected modality-specific spatial estimates (visual cortex for visual location; auditory cortex for auditory location), posterior parietal regions expressed signatures consistent with reliability-weighted integration, and more anterior parietal areas tracked final perceptual estimates predicted by Bayesian causal inference. Together, these results provided an explicit mapping from computational components—unisensory estimation, cue integration, and causal-structure-dependent estimation—onto distinct cortical stages.

Follow-up electroencephalography (EEG) work from the same group further established the temporal ordering of these computations [58]. Neural signals associated with unisensory estimates emerged earlier than signals consistent with fused estimates, which in turn preceded activity patterns reflecting causal-inference-dependent estimates. This sequential timing complements the spatial hierarchy observed in fMRI and supports the view that causal inference is implemented through staged transformations rather than a single, instantaneous computation [38].

**Figure 2:**
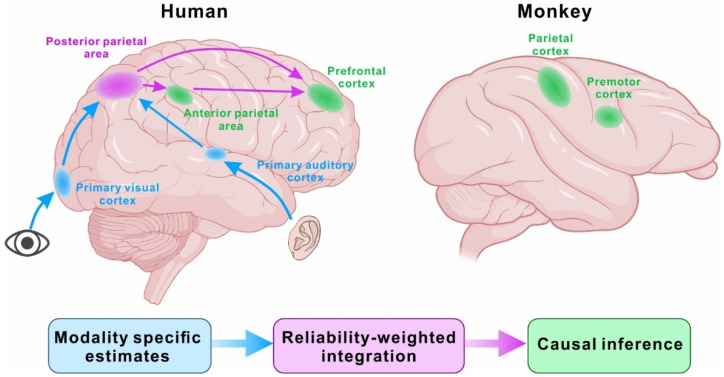
Distributed and hierarchical neural architecture for multisensory causal inference. Proposed cortical hierarchy supporting multisensory causal inference, integrating evidence from human neuroimaging and non-human primate neurophysiology. In humans (**left**), early sensory cortices encode modality-specific estimates (e.g., primary visual and auditory cortex), parietal regions support multisensory integration, and more anterior parietal and/or prefrontal regions are implicated in representing causal-structure-dependent estimates and/or causal decisions [16,58]. In monkeys (**right**), causal-inference-related signals have been linked to premotor and parietal circuits [56,57].

Complementary evidence comes from magnetoencephalography (MEG) during audiovisual frequency judgment [43]. These results likewise revealed hierarchical transformations of sensory representations within parietal cortex. In addition, signals in the prefrontal cortex (PFC) showed the strongest correspondence with model-derived estimates, consistent with a role for frontal regions in evaluating or selecting causal structure (Figure 2). Differences in the prominence of prefrontal involvement across studies likely reflect task demands and analysis choices, emphasizing that the causal-inference network is flexible rather than fixed.

Direct links between causal inference and neuronal activity have begun to emerge from primate electrophysiology. In a virtual-reality visuomotor paradigm, Fang and colleagues systematically manipulated visuoproprioceptive disparity in macaques and observed behavioral biases that increased for small conflicts but saturated at larger conflicts, consistent with a graded shift from integration toward segregation [56,57]. Simultaneous single-unit recordings in the premotor and parietal cortex revealed neural activity that tracked conflict magnitude and covaried with trial-by-trial binding tendencies, providing evidence that frontoparietal neurons carry signals relevant to causal-structure inference [57]. Together with human neuroimaging, these findings support a distributed and hierarchical account: early sensory cortices emphasize modality-specific representations, parietal cortex supports multisensory transformations and integration-related computations, and frontal regions contribute to structure evaluation and selection (Figure 2).

While the cortical “where” and “when” of causal inference are increasingly constrained, the mechanistic “how” remains open: how neural circuits represent uncertainty, estimate common-cause belief, and generate the characteristic nonlinear transition from integration to segregation as conflict increases. Recent computational proposals outline several plausible routes. These proposals can be broadly grouped into (i) explicit causal-structure implementations, in which neural activity explicitly represents and/or infers a variable related to the probability (posterior) of a common cause that mediates the integration–segregation transition, and (ii) implicit computation implementations, in which causal structure is effectively marginalized and causal-inference-like behavior emerges through network dynamics and context-dependent readout without an explicitly represented causal-structure variable.

In explicit causal-structure implementations, mixed neural populations can in principle encode or approximate common-cause structure and thereby gate downstream readouts. In the visual–vestibular heading discrimination, circuit models inspired by congruent and opposite tuning have further shown how mixed populations could support estimating common-cause belief and generating behaviorally realistic integration–segregation patterns [59], although the generality of such motifs may depend on the structure of the encoded variable (e.g., circular variables such as heading). Sampling-based approximations (e.g., importance sampling) provide neurally plausible alternatives to exact analytic inference and may scale more naturally to richer causal-structure spaces [60,61].

In implicit computation implementations, causal inference can arise without an explicit causal-structure representation. Probabilistic population coding (PPC) models provide a principled account of reliability-weighted integration and can produce segregation-like behavior via context-dependent readout or gating [62,63,64]. Network models with parallel unimodal pathways and a multisensory layer can learn nonlinear cue-combination rules through unsupervised exposure to stimulus statistics, reproducing attraction for small disparities and reduced coupling for large disparities [61,65]. Predictive-coding architectures offer a related perspective in which hierarchical message passing and error signals can yield causal-inference-like behavior, and extensions of these frameworks suggest how learning could operate across trials to support recalibration [66,67].

Mechanistic contrasts and current empirical constraints: Existing human fMRI/EEG/MEG studies robustly support a distributed, hierarchical, and sequential transformation from modality-specific estimates to integrated and causal-inference-dependent perceptual estimates, favoring implementations in which computations are staged across cortical levels rather than computed in a single locus [16,43,58]. However, these results do not yet resolve whether causal structure is represented explicitly (as a distinct, decodable variable related to the common-cause posterior) or is computed implicitly via routing/readout and network dynamics. Primate electrophysiology showing trial-by-trial neural correlates of binding tendencies in premotor and parietal circuits provides more direct support for causal-structure-related signals, but causal tests remain limited [57].

Overall, current models converge on the idea that Bayesian causal inference can be approximated using biologically plausible motifs—recurrent interactions, lateral inhibition/excitation, hierarchical pooling, adaptive connectivity, and approximate probabilistic computation—yet multiple implementations remain consistent with existing data. Progress will require computationally constrained experiments that combine model-based predictions with targeted neural measurements and perturbations, and will be particularly informative for understanding how fast inference variables (such as common-cause belief) could gate slower, experience-dependent plasticity [37].

## 3. Multisensory Recalibration

### 3.1. Conceptualizing Multisensory Recalibration Under Causal Inference

Multisensory perception is shaped not only by moment-to-moment inference, but also by learning processes that operate over longer time scales. When discrepancies between sensory modalities are encountered repeatedly, the perceptual system can undergo multisensory recalibration, gradually adjusting the mapping between sensory signals and perceptual estimates [11,12,26,36].

Within a causal inference framework, sensory discrepancies can be interpreted either as noise arising from a common cause or as evidence for separate causes. This distinction has direct consequences for learning. When discrepancies are consistently inferred to reflect a common cause—corresponding to a high posterior probability of a shared source—updating sensory mappings can improve future perceptual accuracy. In contrast, when discrepancies favor segregation, cross-modal recalibration is reduced or suppressed, as the signals are unlikely to reflect the same underlying variable [67].

Accordingly, recalibration is expected to be strongest for small or moderate discrepancies and attenuated for large conflicts, rather than scaling linearly with physical mismatch [68]. Recalibration also operates across multiple time scales, with rapid adjustments following brief exposure and slower changes accumulating over extended experience, consistent with learning driven by inferred causal structure rather than raw sensory error [31,32,69,70].

In the following sections, we review behavioral and neural evidence for multisensory recalibration and consider how recalibration, in turn, can shape subsequent causal inference within an adaptive multisensory system.

### 3.2. Behavioral Evidence for Multisensory Recalibration

Behavioral evidence across multiple domains demonstrates that multisensory recalibration is a robust and systematic consequence of repeated cross-modal discrepancy. Because sensory signals are inherently noisy and rarely perfectly aligned, the perceptual system must continuously adjust internal mappings to maintain coherent perception.

A classic example of cross-modal recalibration is the ventriloquism aftereffect [27,28,71,72,73,74,75]. When auditory and visual stimuli are repeatedly presented with a consistent spatial offset, observers gradually adapt to the discrepancy. Critically, after the visual stimulus is removed, auditory-only localization remains biased toward the previously presented visual location. This persistent aftereffect indicates that auditory spatial representations have been recalibrated through repeated audiovisual exposure, rather than reflecting a transient influence of visual information. Importantly, recalibration strength does not increase monotonically with spatial discrepancy. Instead, it is typically maximal for small to moderate offsets and saturates or declines for larger disparities, suggesting that only discrepancies interpreted as arising from a common source effectively drive learning.

Recalibration is not restricted to external spatial perception but also extends to internal representations of the body. In the rubber hand illusion, synchronous visual and tactile stimulation of an artificial hand and a participant’s hidden real hand can induce a sense of ownership over the artificial limb [29,30,76,77,78]. This illusion is accompanied by a systematic proprioceptive drift toward the visually observed hand, which can persist beyond the period of multisensory stimulation [29]. Such persistence indicates a recalibration of visuo-proprioceptive mappings rather than a purely online multisensory bias. As with audiovisual recalibration, this effect is constrained by spatial and temporal correspondence, weakening when cross-modal consistency is reduced.

**Figure 3:**
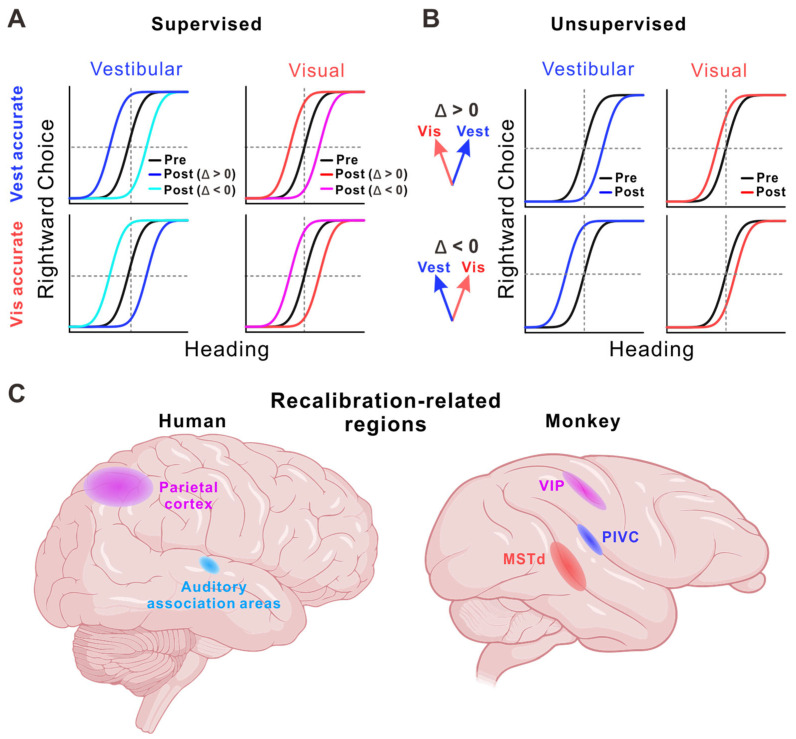
Behavioral signatures and candidate neural substrates of multisensory recalibration. (**A**) Supervised recalibration (with external feedback): visual and vestibular psychometric functions shift in a coordinated (“yoked”) manner to reduce error relative to a feedback-defined reference. (**B**) Unsupervised recalibration (without feedback): psychometric functions shift in opposite directions to reduce internal cross-modal conflict, promoting consistency between cues. Curves depict pre- and post-exposure performance under different conflict directions (Δ > 0 or Δ < 0). (**C**) Candidate brain regions implicated in recalibration across species, including parietal cortex and auditory association areas in humans [81,82] and areas such as ventral intraparietal area (VIP), the dorsal medial superior temporal area (MSTd), and the parieto-insular vestibular cortex (PIVC) in monkeys [83,84].

Multisensory recalibration has been studied in particularly rich detail in the context of self-motion perception, where visual and vestibular cues jointly inform estimates of heading and movement [31,32]. When these cues are placed in sustained conflict, behavioral studies show systematic recalibration of perceptual estimates, revealing plasticity in how visual and vestibular information is combined. Visual–vestibular recalibration can arise through distinct behavioral regimes. When explicit external feedback about perceptual accuracy is available, recalibration is guided by this feedback, leading to coordinated shifts in visual and vestibular estimates that reduce error relative to the external reference [31] (Figure 3A). In contrast, when feedback is absent, recalibration relies on internal consistency. Under these conditions, visual and vestibular estimates tend to shift in opposite directions, reducing cue conflict without reference to an external ground truth [32] (Figure 3B). This unsupervised form of recalibration allows the perceptual system to stabilize performance even in environments where reliable feedback is unavailable.

Together, behavioral findings indicate that multisensory recalibration is strongest when discrepancies are small and consistent with a shared cause, whereas large conflicts that favor segregation constrain or limit learning [26,27,29,31,32,79,80]. These patterns suggest that recalibration reflects learning under causal inference constraints, providing a principled link between moment-to-moment perceptual inference and longer-term sensory plasticity. In the next section, we review neural evidence for multisensory recalibration and examine how recalibration-related plasticity may interact with neural mechanisms of causal inference.

### 3.3. Neural Mechanisms of Multisensory Recalibration

Behavioral recalibration implies that repeated exposure to multisensory discrepancies induce lasting changes in neural representations. Early neural evidence for cross-modal recalibration points to changes in modality-specific sensory representations. Human fMRI studies of audiovisual spatial recalibration have shown that shifts in auditory localization following prolonged audiovisual conflict are accompanied by altered activity in auditory association areas, including planum temporale [82]. These findings indicate that recalibration can be implemented, at least in part, through plastic changes within unisensory cortical representations.

However, recalibration-related plasticity is not restricted to early sensory areas. Human EEG studies indicate higher-order association regions, particularly within parietal cortex. During recalibration phases involving audiovisual discrepancies, parietal activity differs markedly from baseline and is enhanced relative to pre-exposure conditions [81]. These findings suggest that parietal regions may encode discrepancy-related or learning-related signals that guide recalibration. This role is consistent with the broader involvement of parietal cortex in multisensory integration and causal inference, positioning it as a potential interface between moment-to-moment inference and longer-term learning.

Direct evidence for multisensory recalibration at the level of individual neurons comes from classic animal studies of cross-modal plasticity. Experiments in juvenile barn owls demonstrated that sustained alterations of visual input leads to adaptive realignment of auditory spatial maps in the optic tectum, with auditory representations shifting to match the displaced visual field [25,33,34,35]. The strong developmental dependence of this effect—robust in juveniles but markedly reduced in adults—highlights the role of experience-dependent plasticity in shaping multisensory representations, while also motivating the search for mechanisms supporting recalibration in mature systems [25,35].

Further insights into recalibration mechanisms come from studies of visual–vestibular self-motion perception, where neural tuning can be directly related to behavioral adaptation [83,84]. Recordings from the macaque cortex reveal that recalibration critically depends on task structure and feedback availability. During supervised recalibration with external feedback, neuronal tuning in VIP shifts in parallel with behavioral recalibration, whereas tuning changes in MSTd are comparatively modest [83]. In contrast, during unsupervised recalibration without feedback, neural adjustments follow a different pattern: vestibular tuning shifts in the direction of behavioral recalibration, while visual tuning shifts in the opposite direction within VIP, effectively reducing internal cue conflict [84]. Distinct patterns observed across VIP, MSTd, and PIVC further indicate that different cortical regions contribute differentially depending on whether recalibration is driven by external reference signals or by internal consistency (Figure 3C).

At the level of neural computation, candidate accounts differ in whether recalibration reflects primarily error-driven plasticity—in which accumulated sensory errors reshape cue-to-percept mappings—or causal-inference-gated learning, in which learning is modulated by the inferred probability that cues arise from a common cause. The neural evidence reviewed here most directly supports an error-driven component, as recalibration is accompanied by measurable shifts in sensory representations (e.g., altered responses in the auditory association cortex and realignment of spatial maps in the optic tectum) [81,82]. At the same time, several observations indicate that recalibration is not a fixed, purely local update rule: learning depends strongly on task structure and feedback, and tuning changes can differ across areas and modalities under supervised versus unsupervised exposure [83,84]. These features are compatible with the possibility that higher-order processes—potentially related to discrepancy evaluation or inferred causal structure—can modulate when recalibration is expressed and how it is distributed across modalities, but the current evidence remains largely correlational and does not establish a specific gating mechanism.

Together, these findings suggest that multisensory recalibration is implemented through coordinated plasticity across multiple levels of the sensory hierarchy. Sensory cortices express recalibrated representations, while higher-order association areas—overlapping with networks implicated in multisensory integration and causal inference—appear to regulate when and how learning occurs. This distributed organization provides a neural substrate through which experience-dependent recalibration can be flexibly gated by inferred causal structure, setting the stage for a close interaction between recalibration and causal inference.

## 4. Linking Multisensory Causal Inference and Recalibration

**Figure 4:**
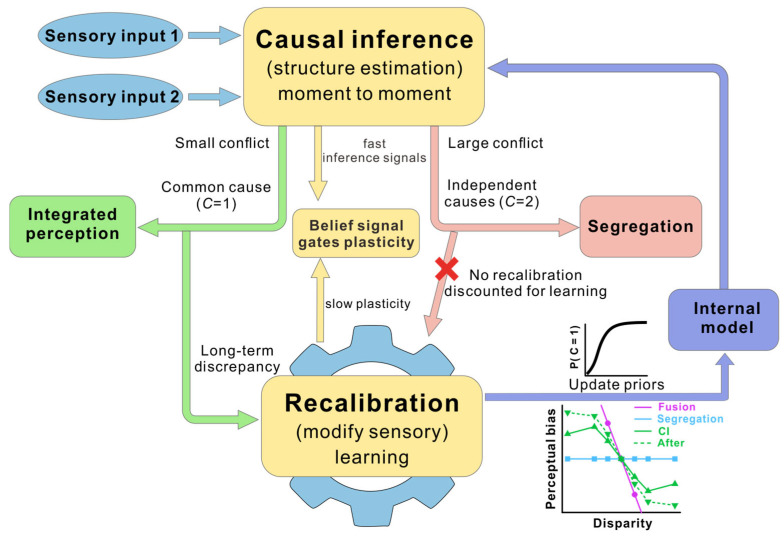
A closed-loop framework linking causal inference and recalibration. Conceptual model illustrating moment-to-moment causal inference and slower multisensory recalibration interact across time scales. Two sensory inputs drive a causal-inference computation. When conflict is small, cues are more likely assigned to a common cause (C=1), supporting integrated perception and allowing discrepancy signals to accumulate over time to drive recalibration. When conflict is large, cues are more likely assigned to independent causes (C=2), promoting segregation and suppressing recalibration. Recalibration updates an internal model (e.g., sensory representations, reliability estimates, or cross-modal correspondence), which feeds back to shape subsequent causal inference by modifying priors and/or likelihoods. Long-term recalibration can reduce the effective discrepancy under the updated mapping, biasing subsequent causal inference toward higher P(C=1) and shifting behavior toward fusion over a broader discrepancy range. A hypothesized neural correlate of this coupling is that trial-wise signals related to inferred causal structure (“belief”) modulate plasticity during exposure, predicting subsequent learning rate and the magnitude of recalibration beyond physical discrepancy alone.

The relationship between multisensory causal inference and recalibration can be understood as an interaction between inference and learning operating across distinct but coupled time scales (Figure 4). Causal inference governs how sensory signals are interpreted on a moment-to-moment basis, whereas recalibration reflects slower adaptive changes that accumulate across repeated experience [67]. Rather than functioning as independent mechanisms, these processes form a closed-loop system that supports both perceptual flexibility and long-term stability.

As illustrated in Figure 4, causal inference operates at a fast time scale to estimate the causal structure underlying current sensory input. By evaluating whether multisensory signals are likely to originate from a common cause or from separate sources, it determines whether cues should be integrated or segregated and generates immediate perceptual estimates. Critically, this inference also determines how sensory discrepancies are interpreted: discrepancies attributed to a common cause are treated as internal noise or bias, whereas discrepancies attributed to separate causes are discounted and do not drive learning [41].

Recalibration emerges when discrepancies that are consistently interpreted under a common-cause hypothesis are encountered repeatedly. In this case, discrepancy-related signals are accumulated over time and drive plastic changes in sensory or multisensory representations [85]. Operating on a slower time scale, recalibration modifies internal mappings rather than immediate perceptual outputs, accounting for the persistence of recalibration effects even when multisensory stimulation is no longer present.

While causal inference constrains when and to what extent recalibration occurs, recalibration in turn shapes future inference by altering sensory representations, reliability estimates, or internal models of cross-modal correspondence [39]. Through this feedback loop, accumulated experience influences the priors and likelihoods that guide subsequent causal judgments [37].

More concretely, recalibration can be formalized as an update to modality-specific internal mappings (i.e., measurement shifts) driven by the discrepancy between a modality’s measurement and its perceptual estimate. Critically, the perceptual estimate itself depends on cue reliabilities, inferred common-cause belief (posterior), and common-cause priors. As a result, long-term recalibration can reduce the effective discrepancy under the updated mapping, such that the same physical discrepancy corresponds to a smaller internal conflict and thus biases subsequent causal inference toward a higher inferred probability of a common cause (higher P(C=1)). In behavioral terms, this predicts that after recalibration the causal-inference curve shifts toward the fusion prediction over a wider range of discrepancies.

To test these ideas directly, we propose longitudinal paradigms in which participants (or monkeys) undergo a recalibration phase with a stable cross-modal discrepancy, followed by a causal-inference test phase that measures both perceptual estimates and integration probability across a range of discrepancies and relative cue reliabilities. In humans, this can be implemented using an audiovisual spatial localization task in which participants additionally provide explicit common-source judgments; in monkeys, an analogous design can be implemented in visual–vestibular heading perception using implicit behavioral readouts of fusion versus segregation. Key experimental manipulations include matching the physical discrepancy distribution across conditions while varying relative cue reliability, training context/priors (e.g., learned co-occurrence statistics or task instructions in humans), and the availability of supervision/feedback during exposure (unsupervised vs. supervised). Key readouts include pre–post changes in the fusion/segregation function (estimated P(C=1) or an equivalent integration index) and changes in perceptual bias/PSE and discrimination thresholds; critically, the direction of pre–post shifts can help dissociate mapping/likelihood updates from prior-level changes in binding tendency.

Neural evidence directly linking causal inference to recalibration is currently more limited than that for causal inference alone, highlighting the need for longitudinal circuit-level studies. A testable hypothesis is that the coupling is implemented by interactions between fast, trial-wise inference signals and slower plasticity expressed in sensory representations. Specifically, a neural variable that tracks inferred causal structure during exposure (e.g., a correlate of P(C=1) or binding belief) should predict subsequent learning rate and the magnitude of recalibration across trials, above and beyond physical discrepancy or sensory error magnitude alone. In contrast, sensory and multisensory areas are expected to primarily reflect the expressed outcome of learning (e.g., tuning shifts or remapped correspondences). This framework therefore predicts a dissociation between signals related to inference (which forecast learning) and sites of plasticity (which express recalibration), and it motivates causal perturbation tests in which disrupting candidate inference-related signals during exposure reduces recalibration while leaving immediate sensory encoding relatively intact.

This framework highlights a unifying principle of multisensory perception: inference and learning are tightly coupled but temporally dissociable processes implemented within overlapping neural circuits. Fast causal inference enables adaptive interpretation of sensory input in the present, while slower recalibration ensures that these interpretations remain aligned with persistent environmental statistics. By linking causal inference and recalibration within a common probabilistic framework, the brain achieves a balance between sensitivity to momentary sensory evidence and robustness to noise and transient conflict.

## 5. Conclusions

In this review, we have synthesized behavioral, computational, and neural evidence showing that multisensory causal inference and recalibration are tightly coupled components of an adaptive perceptual system. Rather than operating in isolation, causal inference and recalibration interact across time scales: causal inference governs how multisensory discrepancies are interpreted in the moment, whereas recalibration reflects experience-dependent plasticity that updates internal sensory representations when discrepancies persist. This framework provides a unified account of how the brain balances perceptual flexibility with long-term stability in noisy and dynamic environments.

Despite substantial progress, several important questions remain unresolved. At a representational level, it is still unclear how explicitly the inferred causal structure is represented in the nervous system. While Bayesian causal inference models posit graded posterior probabilities over causal hypotheses, whether these probabilities are explicitly encoded, consciously accessible, or instead reflected only implicitly through perceptual estimates remains unknown.

At the level of learning and development, it remains open to what extent causal inference mechanisms are innate versus learned. Understanding how causal priors emerge, how quickly they adapt, and how they interact with recalibration processes over the lifespan represents an important direction for future research.

The available behavioral and neural evidence supports a systematic association between causal structure inference and experience-dependent sensory plasticity, but does not yet establish a direct causal link between the two. Accordingly, we propose their interaction as a unifying conceptual framework grounded in converging empirical observations rather than as a confirmed computational or neural mechanism. This framework nevertheless yields testable predictions that can distinguish it from accounts in which recalibration is driven solely by physical discrepancy. First, with an identical discrepancy distribution, manipulating common-cause belief (e.g., via learned priors, task instructions, or contextual statistics) should modulate recalibration magnitude and learning rate; discrepancy-only accounts predict no systematic effect under matched discrepancy. Second, neural signals related to inferred causal structure during exposure should predict subsequent learning (trial-to-trial learning rate and aftereffects) beyond physical discrepancy alone; disrupting such belief-related signals should reduce recalibration while leaving immediate sensory encoding relatively intact. Future work integrating behavioral paradigms, neural circuit analysis, and computational modeling will be essential for testing this framework directly.

Applied implications. Beyond basic science, a causal-inference-informed view of recalibration suggests practical levers for shaping adaptive versus maladaptive plasticity in applied settings. In vestibular and multisensory rehabilitation, training protocols that promote a strong common-cause interpretation (e.g., stable spatiotemporal correspondence, graded conflicts, and appropriately structured feedback) may enhance beneficial recalibration, whereas reducing binding when cues likely arise from distinct sources may help prevent inappropriate drift. For sensory substitution and neuroprosthetic training, establishing consistent cross-modal statistics and explicitly reinforcing common-cause mappings could accelerate stable remapping and generalization. In virtual and augmented reality, controlling cue conflicts and the user’s inferred causal structure—by adapting reliability, alignment, and exposure schedules—may optimize adaptation, reduce cybersickness, and minimize persistent aftereffects when returning to the real world.

## Data Availability

No new data were created or analyzed in this study. Data sharing is not applicable to this article.
